# NK1 Receptor Blockade Is Ineffective in Improving Outcome following a Balloon Compression Model of Spinal Cord Injury

**DOI:** 10.1371/journal.pone.0098364

**Published:** 2014-05-23

**Authors:** Anna Victoria Leonard, Emma Thornton, Robert Vink

**Affiliations:** 1 School of Medical Sciences, University of Adelaide, Adelaide, South Australia, Australia; 2 Division of Health Sciences, University of South Australia, Adelaide, South Australia, Australia; Rutgers - New Jersey Medical School, United States of America

## Abstract

The neuropeptide substance P (SP) is a well-known mediator of neurogenic inflammation following a variety of CNS disorders. Indeed, inhibition of SP through antagonism of its receptor, the tachykinin NK1 receptor, has been shown to be beneficial following both traumatic brain injury and stroke. Such studies demonstrated that administration of an NK1 receptor antagonist reduced blood-brain-barrier permeability, edema development and improved functional outcome. Furthermore, our recent studies have demonstrated a potential role for SP in mediating neurogenic inflammation following traumatic spinal cord injury (SCI). Accordingly, the present study investigates whether inhibition of SP may similarly play a neuroprotective role following traumatic SCI. A closed balloon compression injury was induced at T10 in New Zealand White rabbits. At 30 minutes post-injury an NK1 receptor antagonist was administered intravenously. Animals were thereafter assessed for blood spinal cord barrier (BSCB) permeability, spinal water content (edema), intrathecal pressure (ITP), and histological and functional outcome from 5 hours to 2 weeks post-SCI. Administration of an NK1 receptor antagonist was not effective in reducing BSCB permeability, edema, ITP, or functional deficits following SCI. We conclude that SP mediated neurogenic inflammation does not seem to play a major role in BSCB disruption, edema development and consequential tissue damage seen in acute traumatic SCI. Rather it is likely that the severe primary insult and subsequent hemorrhage may be the key contributing factors to ongoing SCI injury.

## Introduction

Spinal cord injury (SCI) remains a major cause of disability within society, frequently affecting individuals in the prime of their life. To date, therapies have very limited efficacy in attenuating any resultant functional deficits, and accordingly, novel therapeutic approaches are urgently required. SCI is characterized by both primary and secondary injury mechanisms. While the primary injury is clearly irreversible, secondary injury mechanisms are considered reversible and are thus targeted for potential therapeutic interventions. Edema is one of the major secondary injury mechanisms in CNS injury, being considered to significantly contribute to further potentiation of injury development and tissue damage. The development of edema following SCI has been well characterized both within the injury epicentre [1], [2], [3], [4], [5], [6] and in the adjacent segments where a delayed rostrocaudal spread of edema has been demonstrated with time [7], [8]. Such edema may be both vasogenic and cytotoxic in nature, however it has been hypothesized that the initial edema is predominantly vasogenic in nature given that blood-spinal cord-barrier (BSCB) disruption is also present [1], [2], [9], [10], [11]. Importantly, increased edema following injury may lead to raised intrathecal pressure (ITP) [12], which in turn can result in greater tissue damage, and ultimately profound functional deficits.

Neurogenic inflammation has recently been shown to play an important role in the development of edema following a range of CNS injuries [13], [14], [15], [16], [17], [18], [19], [20]. Neurogenic inflammation is a response of perivascular, unmyelinated afferent nerve fibres to injury or infection and is typically characterized by vasodilation, protein extravasation and edema [13], [14], [21]. The vascular response is facilitated by the release of neuropeptides such as substance P (SP) and calcitonin gene related peptide (CGRP). SP is known to preferentially bind to the tachykinin NK1 receptor, activation of which results in increased barrier permeability and edema development [14]. Increased SP immunoreactivity has been associated with increased blood brain barrier (BBB) permeability and edema development following both TBI [13], [17] and stroke [16], whilst antagonism of the NK1 receptor has been shown to reduce BBB permeability and edema, as well as improve functional outcome [13], [17], [22]. Furthermore, our recent investigation following SCI has demonstrated that SP stores are reduced following injury, indicative of SP release, whilst NK1 receptor immunoreactivity increased [20]. Such results implicate a role for SP as a mediator of neurogenic inflammation following SCI. However, whether inhibition of SP may similarly produce a neuroprotective effect following SCI has not yet been investigated.

Accordingly, the current study investigates the effect of administration an NK1 receptor antagonist following a balloon compression model of SCI. Specifically, this paper will assess SP immunoreactivity, BSCB permeability, edema, ITP, histological outcome, and functional outcome from 5 hours to 2 weeks post-SCI. We have utilized the balloon compression model of SCI due to its closed nature, thus facilitating development of increased ITP, and its replication of key primary injury mechanisms in clinical SCI as previously reported [20], [23], [24], [25].

## Materials and Methods

All experimental protocols were conducted according to the guidelines established by the National Health and Medical Research Council and were approved by the animal ethics committees of the University of Adelaide (M-2010-140) and the Institute of Medical and Veterinary Sciences (98/10), Adelaide, South Australia.

### 2.1. Balloon compression model of SCI

New Zealand white rabbits (n = 88) were subject to a balloon compression spinal cord injury as previously described [26], [27]. Briefly, during the 12-hour day cycle, animals were removed from their home cages and anesthetized via a subcutaneous injection of Ketamine (2.5 mg/kg) and Domitor (0.25 mg/kg) mixture. Once a surgical level of anesthesia was achieved, the animal was placed onto a thermostatically controlled heating pad in the prone position. Initially, the dorsal surface of the animal's back was shaved and a midline incision of approximately 10 cm in length was made along the spinous processors (approximately T11 – L2). Paraspinal muscles were retracted and a laminectomy performed. A balloon catheter (ApexTM MonorailTM 4 mm×8 mm, Boston Scientific) was then advanced approximately 4 cm to T10 and rapidly inflated using an inflation device (Boston Scientific) to 8 atm of pressure. The balloon remained inflated for a 5-minute period before being deflated and removed. The muscular wound was sutured closed followed by closure of the skin with surgical clips. An additional group of animals were subject to all surgical procedures except inflation of the balloon catheter (sham; surgery controls).

### 2.2. n-acetyl L-tryptophan (NAT)

Animals were randomly assigned to receive the NK1 receptor antagonist, n-acetyl tryptophan (NAT), or equal volume vehicle (0.9% saline). NAT was administered intravenously at 30 minutes post-SCI at 2.5 mg/kg, with this optimal dose of NAT having been previously determined in studies conducted in our laboratory [28]. For studies with survival times greater than 24 hours, 2 additional i.v. doses were administered daily on day 1 and 2 post-SCI.

### 2.3. BSCB permeability

The Evan's Blue (EB) dye extravasation method, as previously described [14], was used to assess BSCB permeability at 5 hours (vehicle, n = 5; NAT, n = 6) post-SCI (or sham, n = 5). Briefly, 30 minutes prior to perfusion, EB was injected intravenously. The animals were then saline perfused and the spinal cord dissected and 10 mm segments cut. Each segment was homogenized and the absorbance of the supernatant was measured at 610 nm using a spectrophotometer. The level of extravasated EB within each tissue sample was then determined using a previously obtained EB standard curve, and was expressed as ug/mg of spinal cord tissue.

### 2.4. Edema measurement

Animals were assessed for edema at 3 days post-SCI (NAT and vehicle; n = 4/group) or sham (n = 5) using the wet weight/dry weight method as previously described [14]. Briefly, animals were administered a lethal injection of pentobarbital and the spinal cord rapidly removed. The spinal cord was cut into 10 mm segments and the wet weight was obtained. Spinal cord segments were then oven dried at 100°C for 48 hours before the dry weight was measured. The percentage of tissue water content was then calculated using the equation: % Water Content  =  ((Wet Weight – Dry Weight)/Wet weight) X 100

### 2.5. Intrathecal pressure measurement

Animals (vehicle, n = 6; NAT, n = 5; sham, n = 5) underwent a tracheotomy and the right and left femoral artery were dissected. The right femoral artery was cannulated and connected to a syringe pump containing saline, which was administered at 2 ml/h except when taking a blood sample for blood gas analysis. A Codman MICROSENSOR ICP probe was inserted into the left artery to monitor blood pressure. Following balloon compression, a Codman MICROSENSOR ICP probe was introduced into the intrathecal space and extended to the injury epicentre. The Codman probes were connected to an 8 channel Powerlab system (AD instruments) and the output was viewed live and recorded with Labchart (AD instruments). Intrathecal pressure was monitored for a 5 hour period. Blood pressure and blood gases were monitored to ensure physiological parameters were maintained.

### 2.6. Functional outcome

Animals were randomly assigned to sham, vehicle or NAT treated groups (n = 6 per group) and were assessed on days 3, 7, 10 and 14 post-SCI for both sensory and motor outcome. A modified Tarlov Score [23] was used to assess motor function of the hindlimbs. The criteria were as follows: 0 =  No movement; 1 =  minor movement of the hind limb joints; 2 =  major movement of the hind limb joints; 3 =  able to stand properly but unable to hop; 4 =  able to hop, but not properly; 5 =  normal movement. Animals were scored following a recorded 5-minute monitoring period. In addition, a forelimb to hindlimb ratio was calculated by recording the number of hindlimb movements per the first 20 forward moving forelimb steps. This ratio was then converted into a percentage.

The sensory outcome test involved applying the tip end of Dumont #4 fine forceps to the shaved plantar surface of animal's hind paw and assessing the withdrawal time. Animals were placed into an enclosed plastic box with a wire bottom with food provided as a distraction. The fine forceps were then applied to each hind paw 10 times with a 30 second interval between each application. The withdrawal response of the animals to the stimulus was graded as either; 0 =  no response, 1 =  weak response (slight movement of one joint of the hindlimb), 2 =  moderate response (extensive movement of 2 or more joints of the hindlimb), or 3 =  normal response. A sensory score out of 30 was given to each hind paw.

### 2.7. Histological outcome

Animals were randomly assigned to sham (n = 11), vehicle (n = 23) and NAT treated groups (n = 21) and were assessed for histological outcome using immunohistochemical techniques. Briefly animals were perfuse fixed with 10% formalin at 5 hours, 24 hours, 3 days, or 2 weeks post-SCI. Spinal cord tissue was then processed, embedded in paraffin and 5 µm cross sections were cut for assessment of morphological features (H&E stain), SP (Santa Cruz Sc-9758; 1∶2000, EDTA retrieval), NK1 receptor (Advanced Targeting Systems #AB-N33AP; 1∶4000, Citrate retrieval), Albumin (Cappel #0113-0341; 1∶20,000), microglia (Griffonia simplicifolia – Sigma L2140; 1∶200), and AQP4 (Abcam Ab9512; 1∶200, Citrate retrieval).

All sections underwent a similar immunohistochemical procedure, with all antibodies incubated at room temperature and PBS washes applied between each antibody. Briefly, sections were de-waxed, dehydrated and placed in methanol with 30% hydrogen peroxide. Specified microwave antigen retrieval was performed as required and sections incubated for 45 min in 3% normal horse serum. Primary antibody was added overnight before specific biotinylated secondary antibody (Vector,1∶250) was added for 30 minutes. Tertiary streptavidin peroxidase conjugate (SPC; Pierce, 1∶1000) was added for 1 hour and the immunocomplex visualised using 3,3′diaminobenzidine (DAB; Sigma) as a chromogen in the peroxidase reaction.

All sections were scanned at high resolution using the Hamamatsu Nanozoomer. Slides were viewed using the associated proprietary viewing software (NDP.view v1.1.27, Hamamatsu). Qualitative assessments were made by a blinded assessor using a ranking system (0 =  no staining to 10 =  extensive dark staining). Alternatively, whole sections were exported and assessed using our previously published color deconvolution method [29].

### 2.8. Statistical Analysis

All statistical tests were undertaken using GraphPad PRISM®. Evan's Blue extravasation, edema, ITP, Plantar prick test, and color deconvolution were analyzed using a two-way analysis of variance (ANOVA) followed by Bonferroni post-tests, with data expressed as mean ± standard error of the mean (SEM). Immunohistochemistry ranking and modified Tarlov score were analyzed using the Kruskal Wallis ANOVA followed by Dunn's multiple comparisons test. This data was expressed as the median and interquartile range (immunohistochemical ranking) or as the median and individual data points (Tarlov score).

## Results

### 3.1. BSCB Permeability– Evan's Blue extravasation

**Figure 1:**
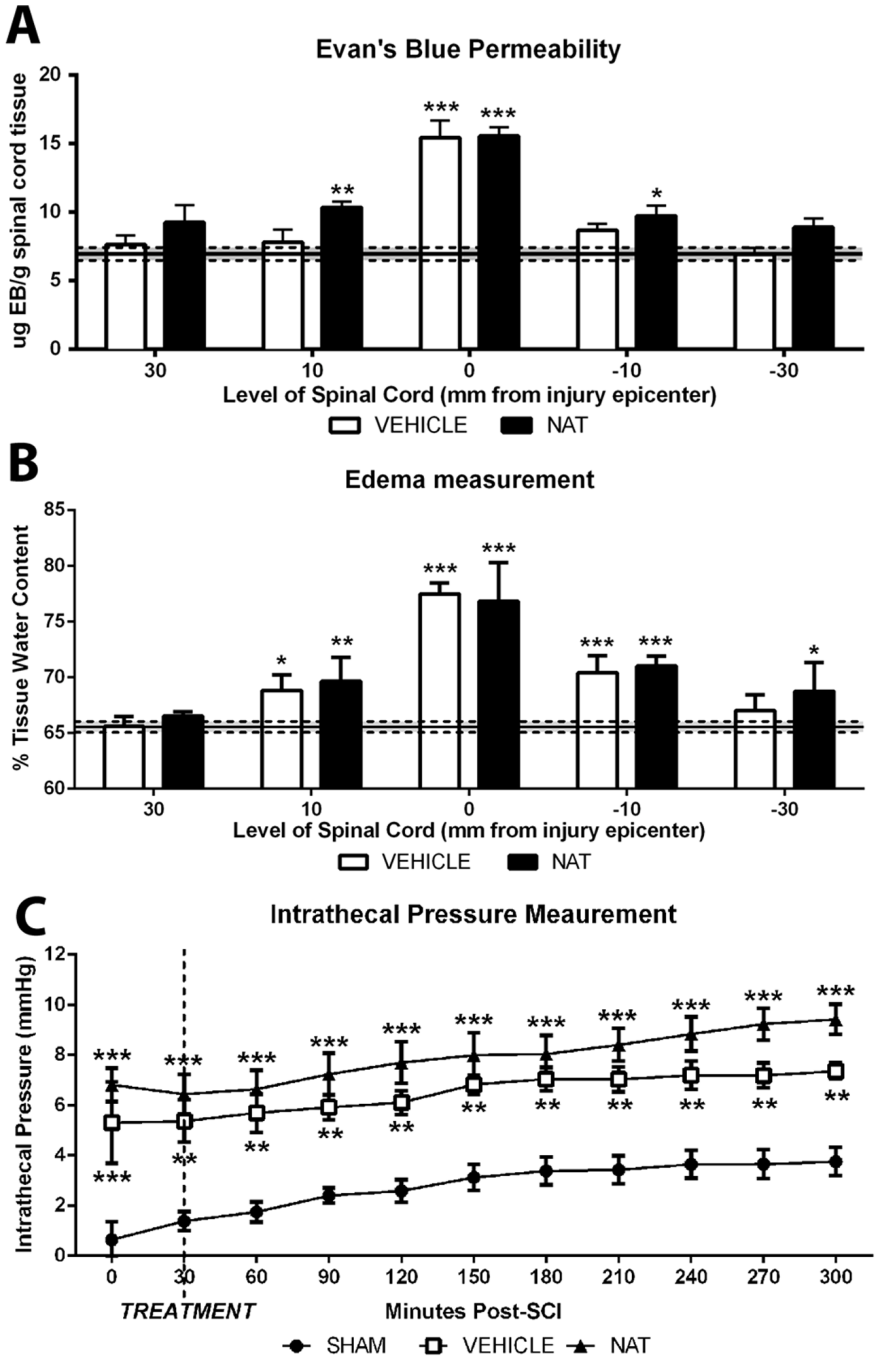
The effect of NAT administration on barrier permeability at 5-SCI was determined by the extent of EB extravasation (A). The percentage of spinal cord tissue water content was measured to determine the extent of edema development at 3 days post-SCI (B). The effect of NAT administration on ITP was measured for a 5 hour monitoring period (C). Sham levels indicated by the dashed line in (A) & (B). *denotes p<0.05, ** denotes p<0.01, *** denotes p<0.001 compared to sham.

Sham animals demonstrated minimal EB extravasation with an average of 6.93±1.25 µg EB/g tissue measured along all segments of the spinal cord (Figure 1A). After injury, a significant (p<0.001) increase in EB extravasation to 15.43±3.04 µg EB/g tissue was observed in vehicle treated animals within the injury epicentre at 5 hours post-SCI, with adjacent segments recording similar values to sham. NAT treated animals had similar EB extravasation to vehicles within the injury epicentre (p<0.001), however recorded significantly greater EB values (0.01<p<0.05) in the immediate adjacent segments when compared to shams. No significant differences were seen between vehicle and NAT treated groups.

### 3.2. Edema measurement

The spinal cord tissue water content of sham animals was 65.55±2.08% (Figure 1B). By 3 days post-SCI in vehicle treated animals, spinal cord water content had significantly increased (p<0.001) to 77.47±1.00% in the injury epicentre and to 68.82±1.39% and 70.4±1.56% within the immediate rostral (p<0.05) and caudal (p<0.001) segments, respectively. NAT treated animals had a similar edema profile to vehicle treated animals at this time, although a greater increase was apparent within the most distal caudal segment of the spinal cord, which was significant compared to sham (p<0.05).

### 3.3. Intrathecal pressure measurement

The intrathecal pressure of sham animals was 0.65±1.61 mmHg at the beginning of the recording period then stabilized and reached 3.75±1.26 mmHg by the end of monitoring (Figure 1C). An immediate significant increase in ITP was observed following injury with vehicle treated animals recording 5.31±3.98 mmHg (p<0.001) at 30 min post-SCI. ITP continued to gradually rise, reaching a maximal ITP of 7.36±0.79 mmHg by the end of 5 hour monitoring period (p<0.001). A similar significant increase in ITP was observed at the beginning of the monitoring period within the NAT treated group, with a recorded ITP measurement of 6.82±1.48 mmHg (p<0.001). Thereafter, a gradual increase in ITP was observed in the NAT treated group reaching a maximal recording of 9.42±1.34 mmHg at the end of the monitoring period (p<0.001). Whilst the NAT treated animals appear to trend slightly higher, no significant difference was observed between treatment groups over time.

### 3.4. Functional outcome

#### 3.4.1. Motor function - Modified Tarlov Scale

**Figure 2:**
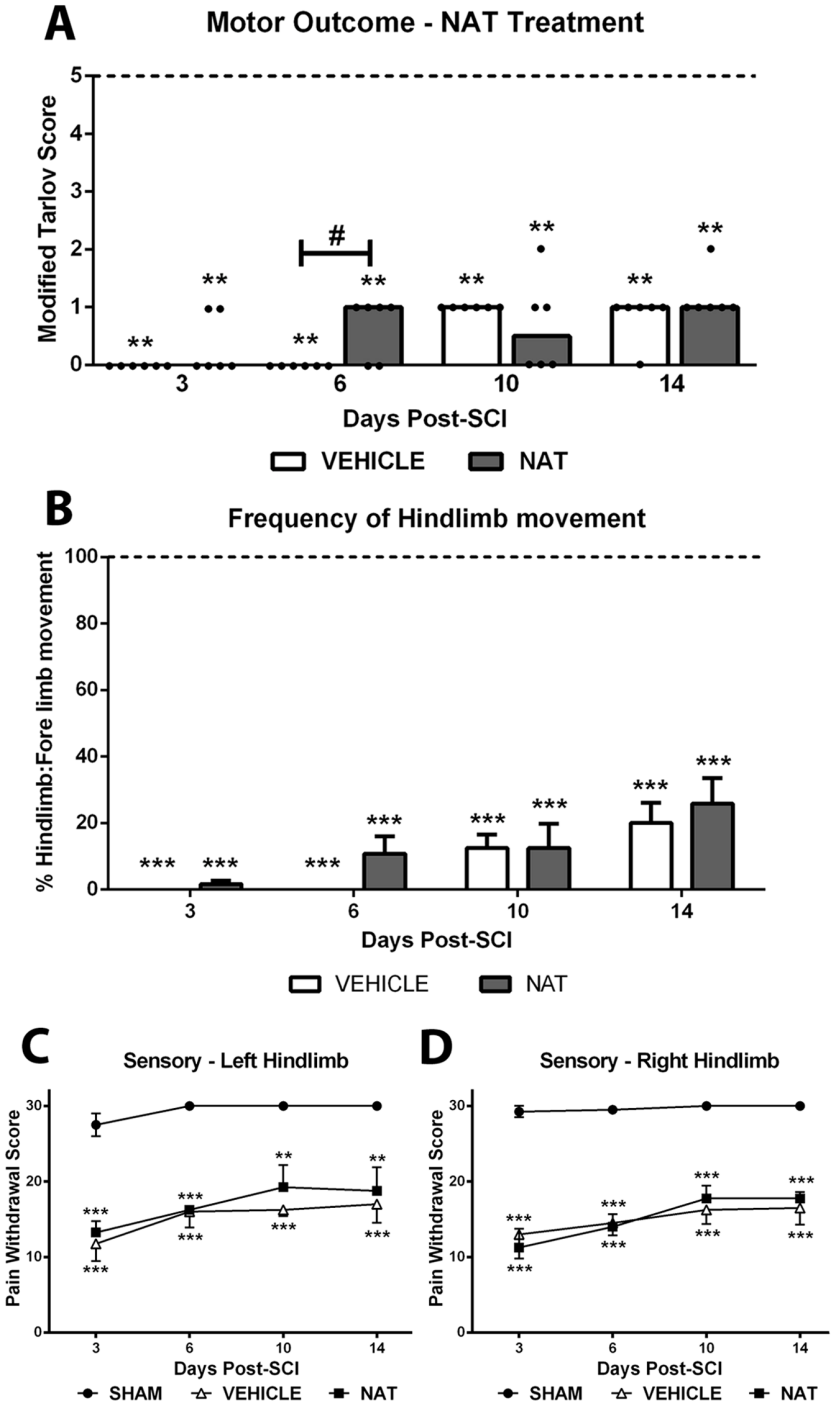
The effect of NAT administration on motor and sensory function following SCI. A modified Tarlov score was used to assess crude motor function (A), data is expressed as the median with individual data points plotted. Whilst a significant improvement was observed at 6 days post-SCI following NAT administration, this improvement was not significantly different to vehicle on days 10 and 14 post-injury. The frequency of hindlimb movement gradually increased in both treatment groups over time (B). Whilst NAT treated animals demonstrated earlier increases in movement frequency, no significant difference was observed between groups. A similar significant decrease in sensory function was observed in both the left (C) and right (D) hindlimbs of both treatment groups. ** denotes p<0.01, *** denotes p<0.001 when compared to sham. # denotes p<0.05 compared to vehicle.

Sham animals demonstrated normal motor function on all assessment days and ranked 5 (Figure 2A). Following injury, vehicle treated animals demonstrated a significant decrease in hindlimb motor function with severe paralysis observed on days 3 and 6 post-SCI. By day 10 and 14, vehicle treated animals ranked 1 and had regained some motor function. In contrast, NAT treated animals had recovered some minor hindlimb joint movement on day 6 post-SCI and accordingly had a significant improvement when compared to vehicle treated animals (p<0.05). However, from day 10 onwards, vehicle and NAT treated animals both had only minor motor function as assessed by the Tarlov score.

#### 3.4.2. Motor function - hindlimb to forelimb ratio

The frequency of the hind limb movement was recorded as the number of hindlimb movements per 20 forelimb steps (Figure 2B). Sham animals demonstrated normal movement with every forelimb step followed by a hindlimb step, resulting in a 100% normal hindlimb movement. Vehicle treated injured animals showed no hind limb movement on day 3 and subsequently recorded 0% for the frequency of hind limb movement. Thereafter, a gradual increase in the frequency of hindlimb movement was observed, reaching 20±15.17% by day 14 post-SCI. As in the Tarlov score, NAT treated animals demonstrated earlier increases in the frequency of hindlimb movement with 10.83±12.81% recorded on day 6 post-SCI, and had a higher maximal frequency of movement of 25.83±18.82% observed on day 14 post-SCI. However, the improvement in hindlimb motor function produced by NAT treatment was only slight and was not significant.

#### 3.4.3. Sensory function – prick test

Sham animals demonstrated normal sensory function over the 14 day assessment period, with only a slight deficit observed on day 3 in the left hindlimb. Such results suggest that the surgical procedure did not affect sensory function (Figure 2C and 2D). Vehicle treated animals demonstrated a significant decrease on day 3 post-SCI to 11.75±4.57 and 13.00±6.38 for the left and right hindlimbs, respectively (p<0.001). Some spontaneous improvement was observed over the assessment period and by day 14 the pain withdrawal score was 17.00±4.96 and 16.50±4.43 for the left and right hindlimbs, respectively (p<0.001). NAT animals similarly demonstrated a significant decrease in sensory function following injury, obtaining a pain withdrawal score of 13.00±2.98 and 10.00±4.99 for the left and right hindlimbs, respectively, on day 3 post-SCI (P<0.001). Similarly to vehicle treated animals, NAT treated animals recorded a slight spontaneous improvement in sensory function in both hindlimbs during the assessment period recording a pain withdrawal score on day 14 post-SCI of 18±6.24 and 17.5±1.71 in the left and right hindlimbs respectively.

### 3.5. Histological outcome

#### 3.5.1. Morphological features – H&E staining

**Figure 3:**
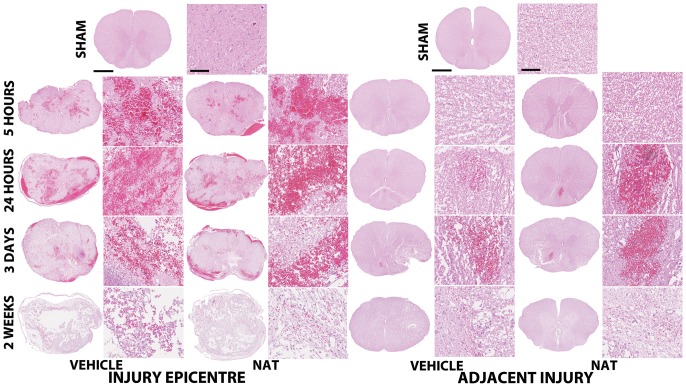
H&E staining demonstrates the morphological changes within the injury epicentre from 5 hours to 2 weeks post-SCI. Hemorrhage was predominant within 24-SCI, with marked tissue loss observed by 2 weeks post-SCI. No differences were observed between vehicle and NAT treatment groups. Cross section scale bar  = 1 mm, high magnification scale bar  = 100 µm.

Sham animals had normal tissue morphology and architecture (Figure 3). SCI resulted in focal areas of severe hemorrhage within the injury epicentre, predominantly within the grey matter, and substantial tissue disruption at 5 hours. The hemorrhage continued to spread and was diffusively located through the injury epicentre by 24 hours. Localized hemorrhage was also observed predominantly within the dorsal aspect of the white matter in the adjacent segments at this time. By 3 days post-SCI, moderate diffuse hemorrhage was observed within the injury epicentre, whilst minor hemorrhage was still apparent within the adjacent segments. Additionally by this time, loss of tissue morphology and architecture are clearly evident in the injury epicentre. By 2 weeks post-SCI extensive tissue loss was evident within the injury epicentre with minimal white matter sparing and minor hemorrhage. The adjacent segments also demonstrated loss of tissue centred within the grey matter and radiating outwards. No differences in morphological features were observed between treatment groups at any time point post-SCI.

#### 3.5.2. Substance P immunoreactivity - Dorsal Horn region

**Figure 4:**
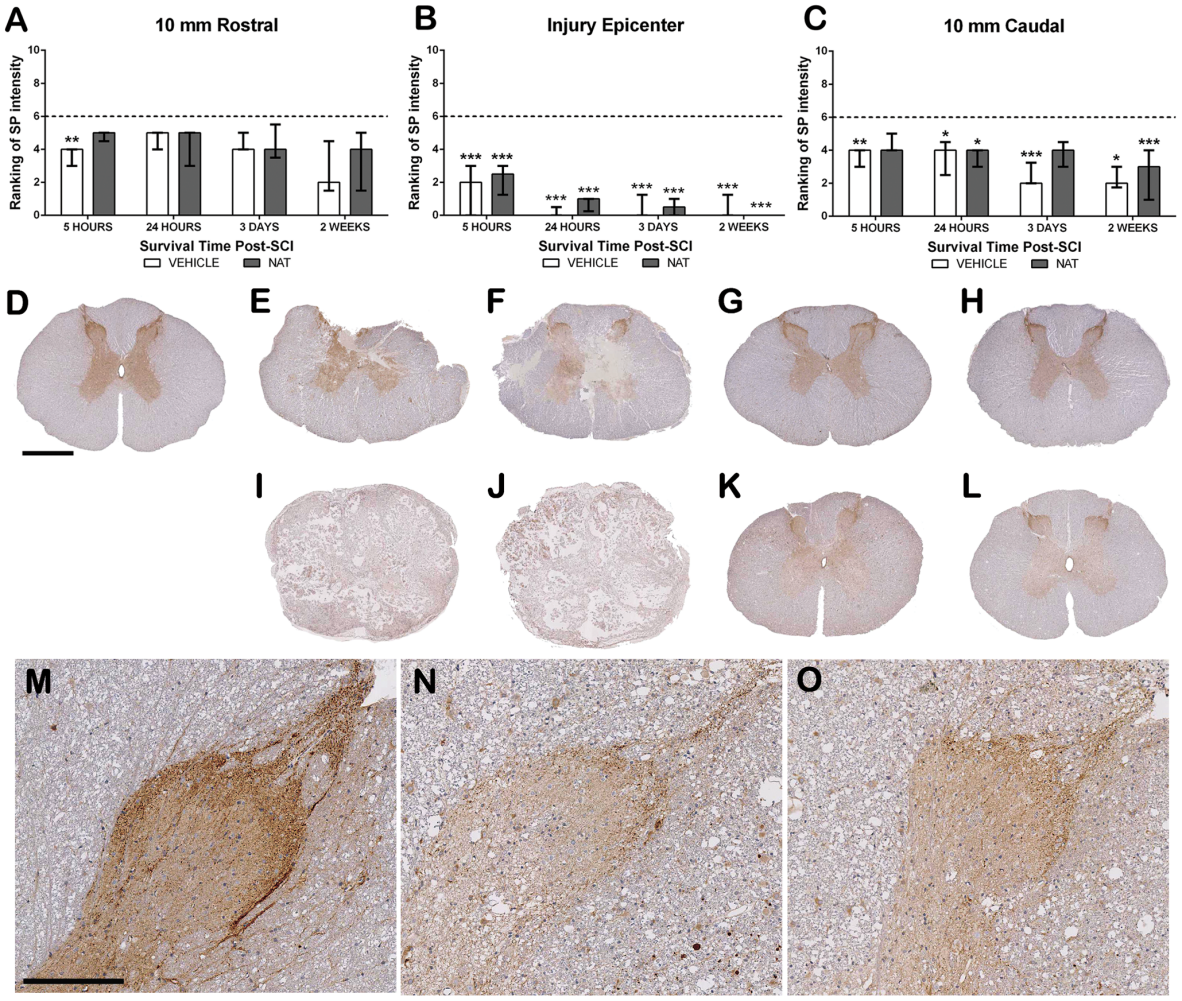
Assessment of the effect of NAT treatment on SP immunoreactivity within the dorsal horn region following SCI. Ranking of SP immunoreactivity within the injury epicentre (B) and at 10 mm rostral (A) and 10 mm caudal (C). Sham sections demonstrated moderate SP immunoreactivity (D). At 5 hours post-SCI reduced immunoreactivity was observed in both vehicle and NAT treatment groups within the injury epicentre (E = Vehicle, F = NAT), whilst a slight reduction was apparent within the adjacent segments (G = Vehicle, H = NAT). However, a significant loss was observed by 2 weeks post-SCI within the injury epicentre (I = Vehicle, J = NAT) and within the adjacent segments (K = Vehicle, L = NAT). Higher magnification images clearly demonstrate this difference (M =  sham, N = 2 week Vehicle adjacent, O = 2 week NAT adjacent). Low magnification scale bar  = 1 mm, High magnification scale bar  = 200 µm. Dashed line (A–C) represents sham median.

Sham animals demonstrated a moderate level of SP immunoreactivity (median  = 6) within the grey matter with particular predominance in lamina I and II of the dorsal horn (Figure 4). Within the injury epicentre, there was a significant decrease in SP immunoreactivity in both treatment groups when compared to sham (p<0.001), with further decreases in both groups at 24 hours (vehicle  = 0 p<0.001; NAT = 1 p<0.01) and 3 days post-SCI (p<0.001 for both groups). By 2 weeks, the injury epicentre in both groups was completely devoid of SP immunoreactivity (p<0.001). The loss in SP immunoreactivity within the adjacent sections was not as pronounced as in the injury epicentre until day 3 in the 10 mm caudal section (median  = 2; p<0.001) and 2 weeks in the 10 mm rostral section (median  = 2). Interestingly at day 3 within the 10 mm caudal section, NAT treated animals had greater SP expression, with a median ranking of 4. Similarly, in the rostral section at 2 weeks, NAT treated animals recorded a higher median ranking of 4 suggesting NAT treatment resulted in greater SP immunoreactivity.

#### 3.5.3. Substance P immunoreactivity - Perivascular region

**Figure 5:**
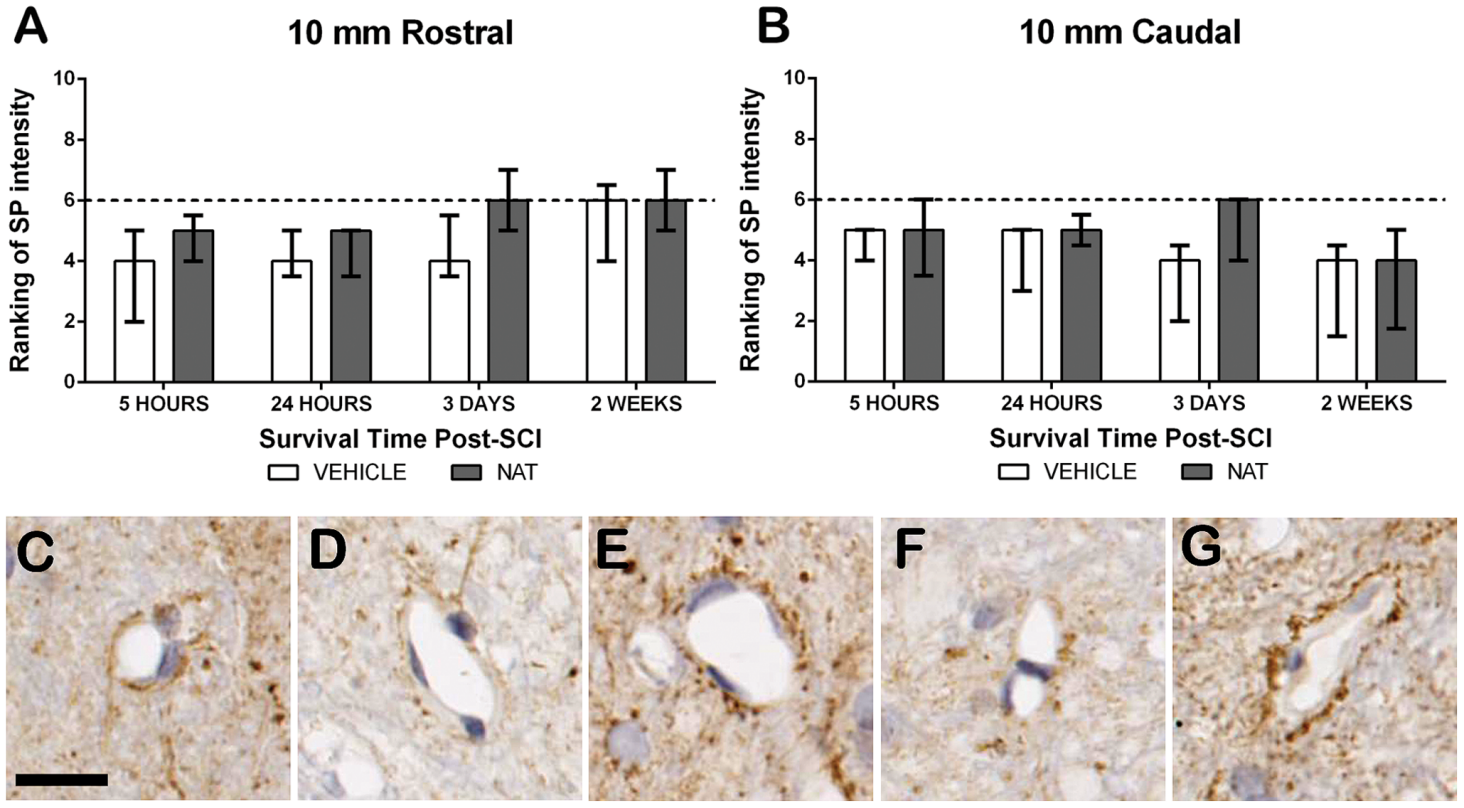
Assessment of the effect of NAT treatment on SP immunoreactivity within the perivascular region following SCI. Ranking of SP immunoreactivity at 10(A) and 10 mm caudal (B) to the injury epicentre. Sham sections demonstrated moderate SP immunoreactivity (C). At both 5 and 24 hours post-SCI a slight decrease was observed within both segments of vehicle and NAT treatment groups. However at 3 days post-SCI NAT treated sections at both 10 mm rostral (E) and caudal (G) demonstrated a return to sham levels whilst vehicle treated remained reduced (D = 10 mm rostral; F = 10 mm caudal). *denotes p<0.05, **denotes p<0.01, ***denotes p<0.001, sham median ranking indicated by the dashed line. Scale bar  = 25 µm.

The perivascular region was assessed within the grey matter of the spinal cord (Figure 5). The injury epicentre could not be examined because of severe tissue disruption. Sham animals demonstrated moderate SP immunoreactivity surrounding the vasculature with a median of 6. At 5 hours post-SCI a slight decrease was observed within both adjacent segments in both treatment groups, with this trend remaining at 24 hours post-SCI. Interestingly in both adjacent segments, by 3 days post-SCI the NAT treated group had returned to sham levels, recording a median ranking of 6. By 2 weeks post-SCI, both treatment groups demonstrated comparable immunoreactivity to sham levels in the rostral segment, though remained below sham levels within the caudal segment. No significant differences were detected between treatment groups at any time post-SCI.

#### 3.5.4. NK1 immunoreactivity - Grey Matter

**Figure 6:**
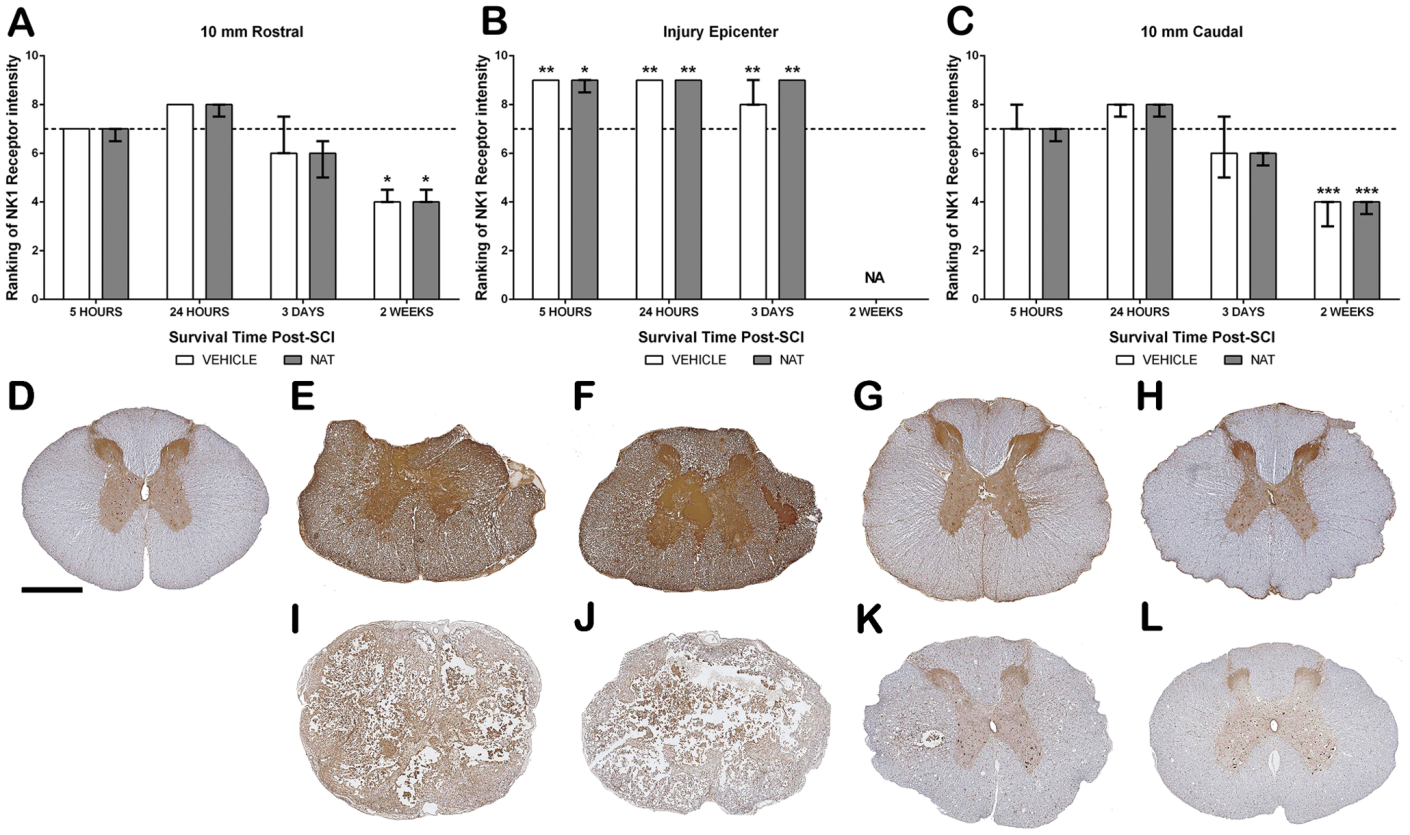
The effect of NAT administration on NK1 receptor immunoreactivity within the grey matter following SCI. Ranking of NK1 receptor immunoreactivity at 10(A), within the injury epicentre (B) and at 10 mm caudal (C). At 24 hours post-SCI a significant increase was observed within the injury epicentre (E =  vehicle and F = NAT), whilst adjacent segments demonstrated a slight increase (G =  vehicle and H = NAT). By 2 weeks post-SCI tissue loss was too great to assess NK1 immunoreactivity within the injury epicentre (I-vehicle, J-NAT) although adjacent segments had reduced immunoreactivity (K-vehicle, L-NAT). No differences were observed between treatment groups at any time point. *denotes p<0.05, **denotes p<0.01, ***denotes p<0.001, sham median ranking indicated by the dashed line (A,B & C). Scale bar  = 1 mm.

Sham sections demonstrated diffuse immunoreactivity within the grey matter with greater intensity observed within the dorsal horn resulting in a median ranking of 7 (Figure 6). At 5 and 24 hours post-SCI within the injury epicentre, a significant increase in NK1 receptor immunoreactivity to a median ranking to 9 was observed within the injury epicentre in both treatment groups (vehicle  =  p<0.01, NAT  =  p<0.05–0.01). Such immunoreactivity remained elevated in both treatment groups on day 3 post-SCI. By 24 hours post-SCI, the adjacent segments also demonstrated a slight increase in NK1 expression centrally within the grey matter in both groups. However by day 3, a slight reduction in NK1 immunoreactivity within these segments was observed in both groups (median  = 5). By 2 weeks post-SCI, the loss of tissue made it difficult to assess NK1 immunoreactivity in the injury epicentre, whereas NK1 immunoreactivity had further decreased in the adjacent segments of both groups, recording a median ranking of 4 (rostral  =  p<0.05, caudal p<0.001).

#### 3.5.5. NK1 immunoreactivity – Perivascular

**Figure 7:**
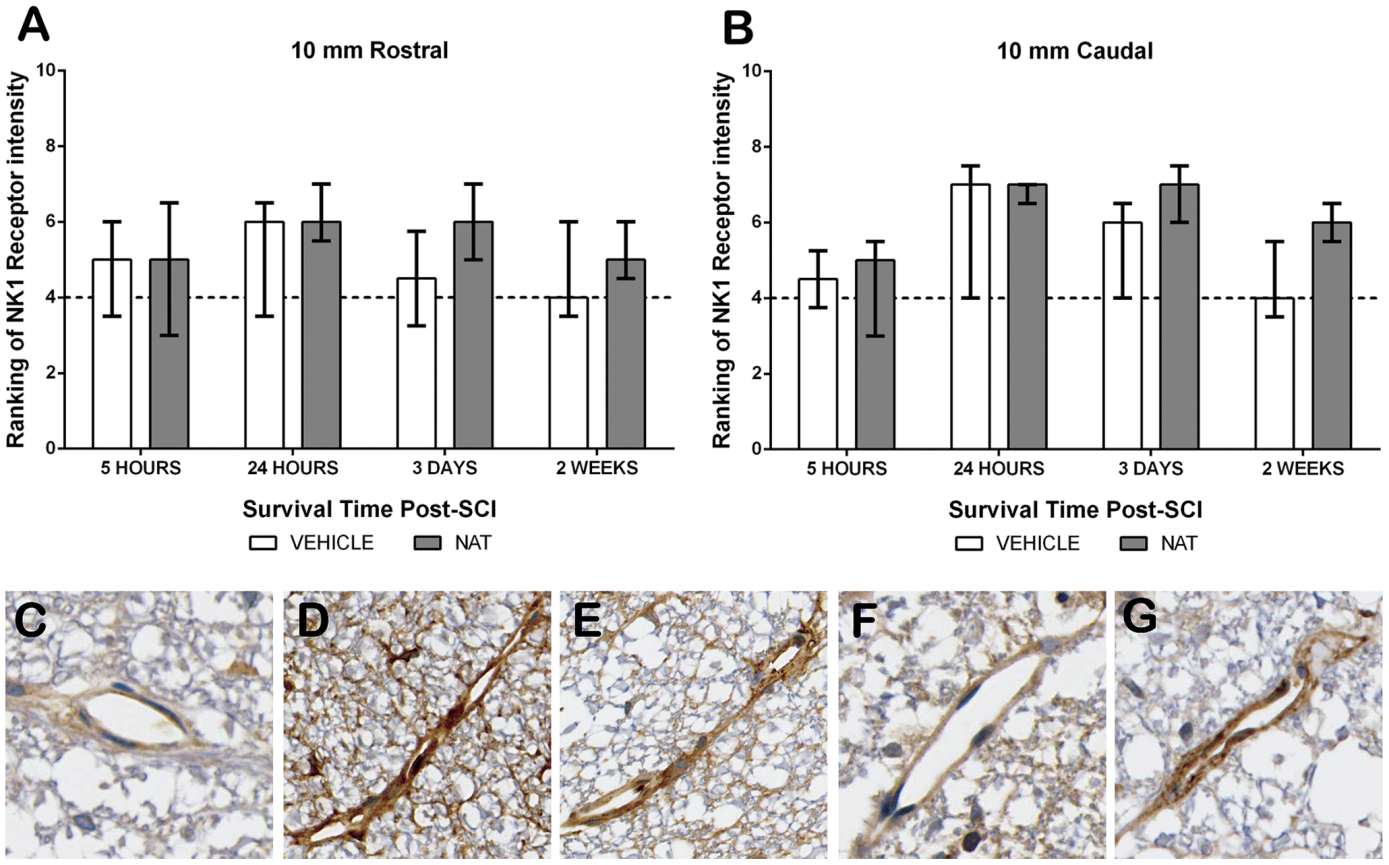
The effect of NAT administration on NK1 receptor immunoreactivity within the perivascular region following SCI. NK1 receptor immunoreactivity ranking at 10(A) and caudal (B). Sham sections demonstrated faint immunoreactivity surrounding the vasculature (C). Notably, at 24 hours post-SCI a marked increase was observed in both vehicle (D) and NAT (E) treatment groups. By 2 weeks post-SCI at 10 mm caudal vehicle treated (F) returned to sham levels, whilst NAT treated remained elevated (G). Median sham ranking indicated by the dashed line (A&B).

Sham sections demonstrated faint NK1 receptor immunoreactivity surrounding the vasculature with a median ranking of 4 (Figure 7). Due to severe tissue disruption, NK1 immunoreactivity was not assessed within the injury epicentre. A slight increase in NK1 immunoreactivity was observed within the adjacent segments of both treatment groups at 5 hours post-SCI. Further increases were observed at 24 hours post-SCI within the caudal segment recording a median ranking of 7 for both treatment groups, whilst the rostral segment was only slightly increased to a median ranking of 6. By day 3, NAT treated caudal segments remained at a median of 7, whereas vehicle treated animals had slightly reduced further to 6, although this was still increased compared to shams. Similarly, within the rostral segment NAT treatment resulted in slightly greater immunoreactivity compared to both vehicle and sham, whereas vehicle treated animals had comparable NK1 immunoreactivity to shams. Vehicle treated animals remained at sham levels at 2 weeks post-SCI in both sections, whilst NAT treatment still recorded above sham levels (rostral median  = 5; caudal median 6).

#### 3.5.6. Albumin immunoreactivity

**Figure 8:**
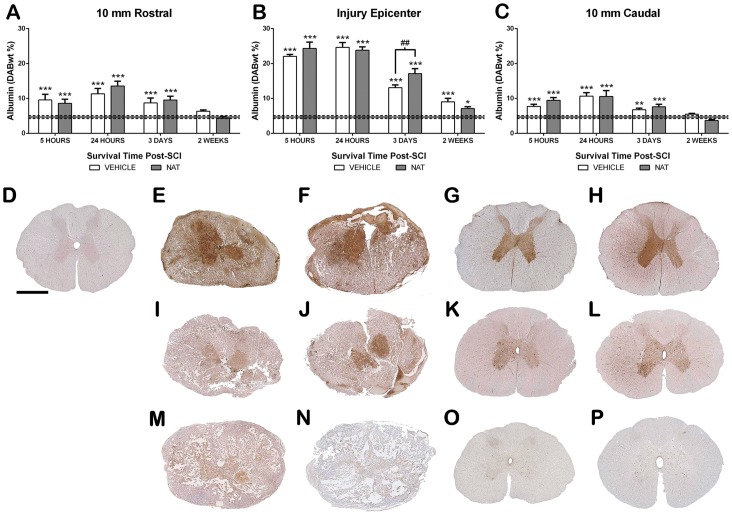
The effect of NAT treatment on albumin immunoreactivity following SCI. Albumin immunoreactivity at 10(A), within the injury epicentre (B) and at 10 mm caudal (C). Sham sections (D) demonstrated minimal immunoreactivity. Immunoreactivity was significantly increased at both 5 and 24 hours post-SCI. Representative images at 24 hours are shown within the injury epicentre (E-vehicle, F-NAT) and adjacent segment (G-vehicle, H-NAT). By 3 days post-SCI albumin immunoreactivity began to reduce within the injury epicentre (I-vehicle, J-NAT) and adjacent segment (K-vehicle, L-NAT), though NAT treatment resulted in significantly greater albumin immunoreactivity when compared to vehicle. Albumin immunoreactivity reduced further by 2 weeks post-SCI within the injury epicentre (M-vehicle, N-NAT), returning to sham levels within the adjacent segments (O-vehicle, P-NAT). *denotes p<0.05, **denotes p<0.01, ***denotes p<0.001 when compared to sham. Mean sham values indicated by the dashed line. Scale bar  = 1 mm.

Albumin immunoreactivity was assessed to quantify the effect of NAT administration on the extent of BSCB permeability following SCI (Figure 8). Sham sections of spinal cord demonstrated minimal albumin immunoreactivity with a DABwt% of 4.7±0.42, indicating that sham surgery did not disrupt the BSCB. Following injury, a significant increase in albumin immunoreactivity was observed within the injury epicentre and became maximal at 24 hours post-SCI reaching 24.64±2.77 DABwt% (p<0.001). Albumin immunoreactivity then decreased over time, although was still significantly greater than shams at 2 weeks (p<0.001). The NAT treated animals had a similar expression of albumin to vehicle treated animals in the injury epicentre, although they recorded significantly greater albumin than vehicle treated animals at day 3 (p<0.05). However by 2 weeks, they had decreased below vehicle treated animals but were still had significantly greater albumin than sham (p<0.05). Similarly, the adjacent segments demonstrated maximal increases at 24 hours post-SCI for both treatment groups (p<0.001), with a decrease over time so that by 2 weeks both groups had returned to sham levels. Moreover, NAT treated animals were comparable to vehicles at all assessment times in the adjacent segments.

#### 3.5.7. Microglial immunoreactivity (ISOB4) - White Matter

**Figure 9:**
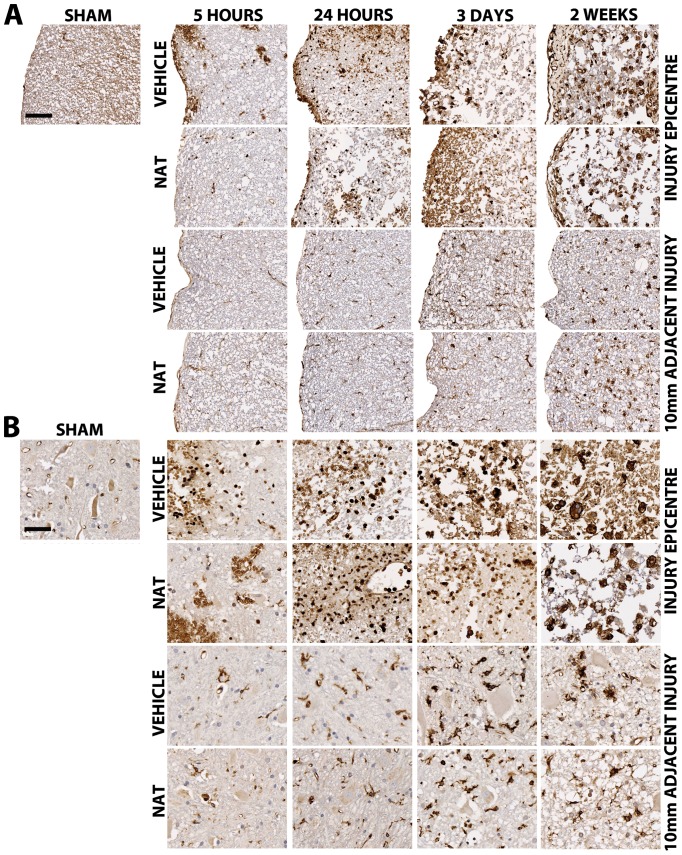
The effect of NAT administration on microglial immunoreactivity following SCI within the white matter (A) and grey matter (B). Sham sections demonstrate minimal microglial immunoreactivity in both the white and grey matter. Within the white matter microglia immunoreactivity is maximal by 2 weeks post-SCI within the injury epicentre and adjacent segment for both treatment groups. Within the grey matter, many small round microglia can be seen within the injury epicentre, increasing in size by 2 weeks post-SCI. Within the adjacent segment of the grey matter many ramified microglia can be seen by 3 days post-SCI and becoming amoeboid in shape by 2 week post-SCI. No differences between treatment groups were observed. Scale bar (A) = 200 µm, (B) = 50 µm.

Sham sections demonstrated low numbers of resting microglia with long fine processes. After injury, small numbers of microglia were observed within the injury epicentre of both treatment groups (Figure 9A). At 24 hours post-SCI many cells with a small round phenotype suggestive of phagocytic activity can be seen. Furthermore, increased numbers of immunoreactive cells can also be seen within the adjacent segments at this time. At 3 days post-SCI, tissue loss is apparent and further increases in immunoreactive cells are seen within the injury epicentre in both treatment groups. By 2 weeks post-SCI greater tissue loss is apparent and florid microglia are present within the white matter, becoming amoeboid in shape by 2 weeks post-SCI. At this time, the adjacent segments of spinal cord also demonstrate increased microglia immunoreacitivty, with many amoeboid cells apparent within the white matter of both treatment groups. No differences in microglia immunoreactivity were observed between vehicle and NAT treated animals.

#### 3.5.8. Microglial immunoreactivity (ISOB4) - Grey Matter

Sham sections again demonstrate low numbers of resting microglia with fine long processes (Figure 9B). At 5 hours post-SCI, hemorrhage is visible in addition to small round immunoreactive cells, suggestive of phagocytic activity, present within the injury epicentre of both treatment groups. At 24 hours post-SCI florid microglia can be seen within the injury epicentre whilst the adjacent segments also demonstrate increased microglia particularly surrounding the blood vessels within both treatment groups. At 3 days post-SCI greater tissue loss was observed within the injury epicentre with many immunoreactive cells still present, and becoming larger in size. The adjacent segments demonstrated numerous activated microglia that appear ramified in nature. By 2 weeks post-SCI increased tissue loss was observed within the injury epicentre with larger phagocytic cells apparent within both vehicle and NAT treated sections. The adjacent segments similarly demonstrate florid microglial activity with many observed as fully ramified and amoeboid in appearance, representing phagocytic activity. No differences in microglial immunoreactivity between vehicle and NAT treated groups were observed.

#### 3.5.9. AQP4 immunoreactivity - Perivascular region

**Figure 10:**
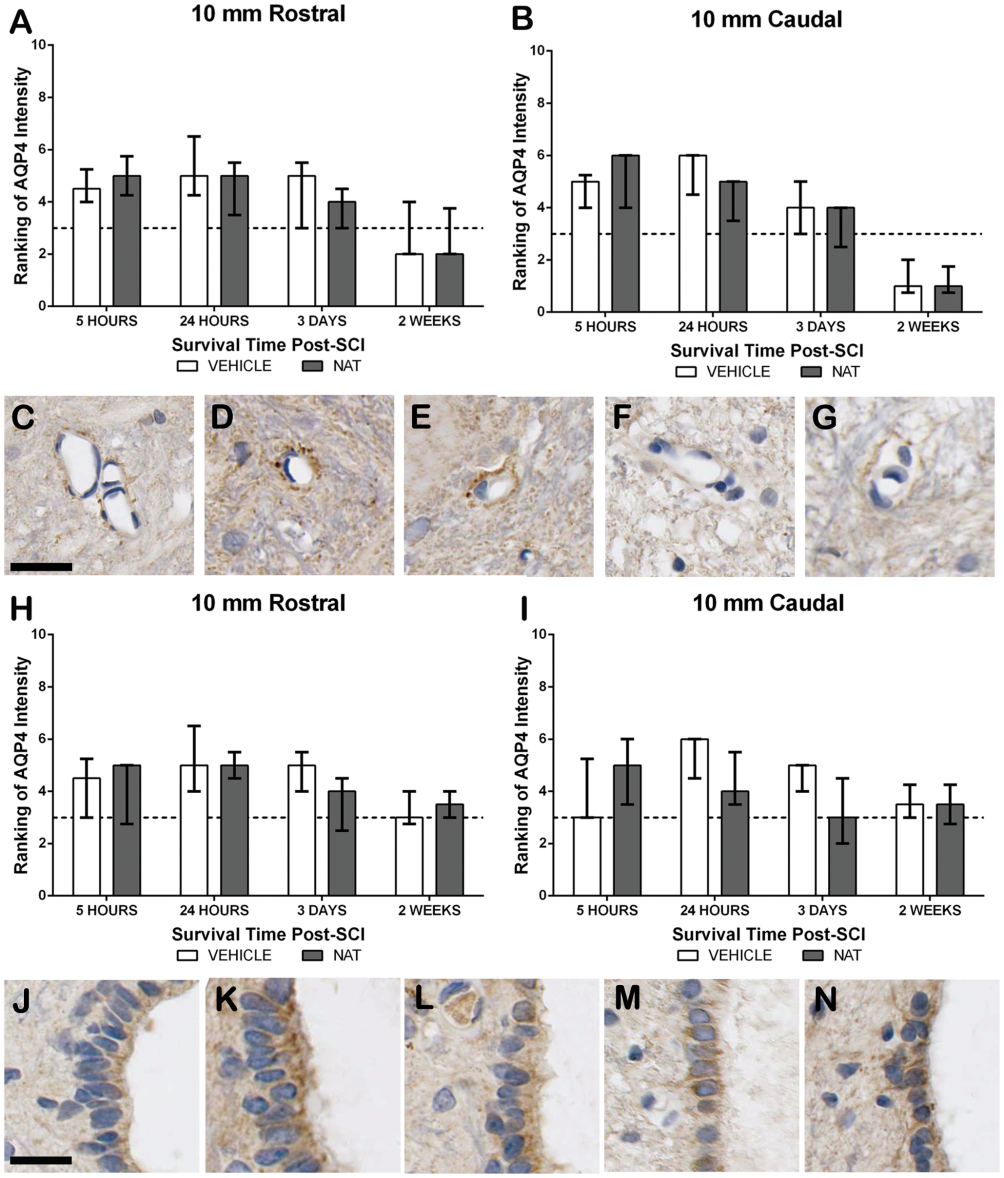
The effect of NAT administration on perivascular and ependymal AQP4 immunoreactivity following SCI. Ranking of perivascular AQP4 immunoreactivity at 10(A) and 10 mm caudal (B). Sham sections demonstrated faint immunoreactivity surrounding the vessels (C). Increased immunoreactivity can be seen at 24 hours following both vehicle (D) and NAT (E) treatment. Reduced immunoreacticity below sham levels was observed at 2 weeks (H-vehicle, I-NAT). Dashed line indicated mean sham values. Ranking of ependymal AQP4 immunoreactivity at 10 mm rostral (H) and 10 mm caudal (I). Sham sections demonstrated faint immunoreactivity within the ependymal cells of the central canal (J). At 24 hours post-SCI increases were apparent, with greater increases in the caudal segments of vehicle treated sections (K) than NAT treated (L). By 2 weeks post-SCI both treatment groups demonstrated comparable AQP4 immunoreactivity to sham levels (M-vehicle, N-NAT). Scale bars  = 25 µm.

Sham sections of spinal cord demonstrated faint AQP4 immunoreactivity surrounding the vasculature within the grey matter, assessed as a median ranking of 3 (Figure 10). Due to severe tissue disruption the injury epicentre was not assessed. Following injury increased AQP4 immunoreactivity was observed by 5 hours and reached a median ranking of 6 by 24 hours post-SCI. A slight decrease was observed at 3 days post-SCI in the caudal section, whereas the rostral section still ranked 6. A further decrease to below sham levels was apparent by 2 weeks (rostral  = 2; caudal  = 1). NAT treatment had a similar pattern of immunoreactivity to vehicle treated animals in the rostral section, apart from on day 3 when they ranked slightly less (median  = 4). Within the caudal section, NAT treated animals recorded slightly higher than vehicles at 5 hours, but then decreased over time so that they were less than vehicles at 24 hours but similar at 3 days and 2 weeks.

#### 3.5.10. AQP4 immunoreactivity - Central Canal region

Sham sections of spinal cord demonstrated faint AQP4 immunoreactivity in the ependymal cells of the central canal with a median ranking of 3 (Figure 10). The injury epicentre was too disrupted to accurately assess AQP4 immunoreactivity. Following injury an increase was observed in the rostral section by 5 hours (median  = 4.5) with a slightly further rise to 5 at 24 hours and 3 days before returning to sham levels at 2 weeks. In contrast, NAT treated animals had maximal AQP4 immunoreactivity in this section by 5 hours (median  = 5), which remained until day 3 when a reduced ranking of 4 was recorded. A further decrease was seen at 2 weeks, although they were still just above shams (median 3.5). Within the caudal section, AQP4 was not increased until 24 hours when a median ranking of 6 was recorded in the vehicle treated animals. AQP4 then declined over time to be near to sham levels by 2 weeks. In contrast, NAT treatment resulted in a increase in AQP4 by 5 hours recording a median ranking of 5, however then declined to 4 at 24 hours and had returned to sham levels by day 3. No significant differences were observed between treatment groups at any assessment time.

## Discussion

The present study has demonstrated that administration of the NK1 receptor antagonist, NAT, does not reduce BSCB permeability, edema, ITP or significantly improve neurological function following SCI. These results suggest that SP mediated neurogenic inflammation does not play a major role in the acute development of such injury processes following traumatic SCI. However, a release of SP was observed, demonstrated by reduced SP immunoreactivity, whilst perivascular NK1 receptor immunoreactivity initially increased before decreasing, which is suggestive of NK1 receptor activation and internalisation. This reflects manifestation of SP mediated neurogenic inflammation, albeit that neurogenic inflammation may not be the predominant driver of these injury processes in the acute phase of SCI.

Although only a bolus dose of NAT was administered on the day of surgery, and for two consecutive days for survival times of 3 days or more, such dosage regimes have previously been used in acute CNS injury with highly beneficial outcomes [13], [17], [22]. Indeed administration of an NK1 receptor antagonist at 30 minutes post-TBI reduced barrier permeability, edema and improved functional outcome, demonstrating that inhibition of SP effects was possible despite a 30 minute delay post-injury [13], [17]. It is unlikely that a shorter timeframe for pharmacological intervention would be clinically relevant. Furthermore, whilst NAT is not barrier permeable, our results demonstrate that the BSCB was disrupted for at least 5 hours post-SCI. Given the severe extent of hemorrhage and destruction of vasculature observed at the site of impact, intravenous administration may have not provided sufficient delivery of NAT. Nonetheless, some differences between vehicle and NAT treated groups were observed in the current study beyond the injury site, indicating that NAT administration was likely successfully delivered centrally. A more direct route of administration, such as an injection into the intrathecal space may provide a more efficient method of administration for future investigations. Given the highly preserved nature of NK1 receptors across different vertebrate species [30], it is unlikely that any lack of effectiveness of NAT treatment was due to low affinity for the rabbit NK1 receptor.

Whilst two isoforms of the NK1 receptor exist, a long complete isoform and a truncated isoform, the binding site of the antagonist is identical in both, and thus the NK1 receptor antagonist used in the current study would be equally effective on both isoforms. However, the long NK1 isoform is known to predominate within the CNS whilst the truncated isoform is most represented within peripheral tissue [31]. Additionally, consideration must be given to related tachykinin receptors, the NK2 receptor and NK3 receptor. Our results do not exclude the possibility that the NK2 or NK3 receptors might play an important role and that inhibition of these receptors may be beneficial to outcome after SCI. However, SP binds preferentially to the NK1 receptor [32], and it is only activation of this receptor that is thought to result in the initiation of neurogenic inflammation. Indeed, It has been demonstrated that the NK2 and NK3 receptors have no direct role in plasma protein extravasation within the CNS [33].

After receiving 3 consecutive daily doses of NAT or saline, NAT treated animals demonstrated a trend for greater perivascular and dorsal horn SP immunoreactivity than vehicle treated animals. Such increases in SP may be explained by the presence of an NK1 autoreceptor, which is thought to regulate SP release through a negative feedback loop [34], [35]. In the current study, blockade of the NK1 autoreceptor due to NAT administration would prevent SP from exerting negative feedback on its own synthesis and release, resulting in greater expression of SP. However, the comparative increase in SP would not have been functional, as blockade of the NK1 receptor with NAT prevents SP from binding and mediating downstream effects.

Interestingly, at 3 days post-injury NAT treatment resulted in significantly higher BSCB permeability compared to vehicle as assessed by albumin. This finding paradoxically implies that inhibition of the NK1 receptor resulted in greater BSCB permeability. Such results are in contrast to previous studies where NAT treatment resulted in reduced BSCB permeability following TBI and stroke [13], [22]. These findings imply that despite similar secondary injury processes arising following SCI, the role of SP may be vastly different in SCI than its role in other acute CNS injuries. One important difference may be the extent of primary mechanical damage, resulting in severe hemorrhage. Previous studies within our group have demonstrated that in severe subarachnoid hemorrhage, NAT administration worsened outcome [36]. These combined results suggest that in models of severe hemorrhage, the primary induced tissue and vasculature damage may dominate over SP mediated neurogenic inflammation in the development of BSCB permeability. Furthermore, as the current study provided evidence that NAT treatment worsened BSCB permeability, SP may actually play a protective role in such severe hemorrhage models. Indeed, a recent study demonstrated that SP treatment promoted a more anti-inflammatory environment following SCI by inducing interlukin-10 and M2 macrophages whilst suppressing nitric oxide synthase and tumour necrosis factor-α [37]. Our own results demonstrate an increase in microglial activity following SCI, though predominantly phagocytic in nature, with no differences between treatment groups. In addition, intrathecal administration of a SP antagonist has been shown to cause a marked decrease in spinal cord blood flow (SCBF) [38] and likely contributed to further damage and BSCB permeability by promoting an ischemic environment. These findings together with the current study suggest that SP may be beneficial following acute SCI.

Regardless of the role of SP following SCI, edema remains a serious complication leading to raised ITP [39], [40], reduced SCBF [9], [40] and myelin damage [1]. Indeed, numerous studies have shown that the extent of edema corresponds to the degree of functional deficits observed following injury [41], [42], [43], [44], [45]. The present study demonstrated increased edema associated with raised ITP following injury, which was maximal at 3 days post-SCI. Furthermore, at this time it was apparent that the adjacent segments, uninjured by the balloon compression, also demonstrated significant increases in edema. Therefore, substantial rostrocaudal spread of edema had occurred following the balloon compression model by 3 days post-SCI. However, albumin immunoreactivity, a marker of BSCB permeability, was maximal at 24 hours post-SCI with a reduction observed at 3 days post-SCI within the adjacent segments. Therefore, significant disparity exists between BSCB permeability and edema formation, indicating that the rostrocaudal spread of edema was not due to BSCB disruption and thus not vasogenic in nature. These results further suggest that neurogenic inflammation is not primarily responsible for the developed edema and may account for the ineffectiveness of NAT administration to reduce edema.

It is possible that the spread of edema may be a compensatory mechanism to reduce the edema present within the injury epicentre. Alternatively, the BSCB may in fact still remain permeable to smaller molecules than albumin. Previous studies have employed alternative smaller tracers of extravasation such as hydrazide [46], horseradish peroxidase [10], [47], [48], protein luciferase [49], and iodine [4], [50] and found greater extension of BSCB permeability. Alternatively, such increases in edema may be due to an ultrafiltration mechanism rather than extravasation as previously described by Nemecek and colleagues [7]. Such ultrafiltration may be aided by reduced blood flow and severe hemorrhage. As NAT treatment has no ability to reduce hemorhage and SP antagonists can markedly reduce SCBF [38], the greater edema within the adjacent segments of NAT treated animals may be due to increased ultrafiltration. Alternatively, in the absence of BSCB disruption, the increased edema may be facilitated by AQP4 water channels.

Indeed, within the current study, AQP4 immunoreactivity was increased following injury at similar times to maximal edema. Although such an increase implies that AQP4 may facilitate edema development, NAT administration resulted in a slight increase in edema and a concurrent reduction in AQP4 immunoreactivity. Therefore, injury induced increases in AQP4 may actually be a compensatory mechanism to assist in fluid clearance. These changes in AQP4 and edema formation contrast to that in TBI, where injury reduced AQP4 expression and NAT treatment restored AQP4 levels, whilst reducing edema [28]. These opposing effects of NAT treatment in TBI and SCI further illustrate the differences in their injury mechanisms. Moreover, these results demonstrate that the relationship between SP and AQP4 warrants further investigation. Taken together, these results demonstrate that AQP4 may play an essential role in the elimination of excess fluid. Indeed, previous investigations have similarly demonstrated that AQP4 plays an integral role in facilitating water clearance following SCI [2], [51], [52]. However, to date, almost all studies of AQP4 following SCI have assessed its function through altered expression [2], [51], or employed AQP4-null mice [12], [52]. As such, further investigation to fully elucidate the role of AQP4 following SCI is required. Ideally, this could be through the pharmacological modulation of the AQP4 water channels, which may then be utilized as a novel therapeutic intervention.

## Conclusions

The current study has demonstrated that despite a release of SP and therefore induction of SP mediated neurogenic inflammation, the severe primary damage that results in destruction of vasculature and hemorrhage may play a greater role in BSCB disruption, subsequent edema development and associated tissue damage and functional deficits following traumatic SCI. Thus, administration of the NK1 receptor antagonist, NAT, did not reduce BSCB permeability, edema, ITP, or improve neurological function following SCI. In contrast, SP may actually play a beneficial role in reducing this ongoing damage associated with traumatic SCI.
